# Descriptive Case Series of Childhood Lymphomas Treated at the Children’s Hospital of Mexico

**DOI:** 10.3390/pediatric18010028

**Published:** 2026-02-10

**Authors:** Miguel A. Palomo-Colli, Daniela Vega-Ruiz, Argelia Escobar-Sánchez, Matilde Galicia-Esquivel, Luis E. Juárez-Villegas, Abigail Morales-Sánchez

**Affiliations:** 1 Hematology and Oncology Department, Hospital Infantil de México Federico Gómez, Mexico City 06720, Mexico; 2 Clinical Research Department, Hospital Infantil de México Federico Gómez, Mexico City 06720, Mexico; 3 Clinical and Experimental Pathology Service, Hospital Infantil de México Federico Gómez, Mexico City 06720, Mexico; 4 Molecular Biology Laboratory, Hospital Infantil de México Federico Gómez, Mexico City 06720, Mexico; 5 Immunobiology and Oncology Research Laboratory, Hospital Infantil de México Federico Gómez, Mexico City 06720, Mexico

**Keywords:** pediatric lymphoma, Hodgkin lymphoma, non-Hodgkin lymphoma, T-cell lymphoma, childhood cancer, ICCC-3 classification

## Abstract

**Background**: Pediatric lymphomas comprise a heterogeneous group of malignancies with substantial variation in their clinical presentation. In Mexico, detailed case-based characterization remains limited. This study summarizes the demographic and clinical characteristics of pediatric lymphomas diagnosed at a national referral center over an 11-year period. **Methods**: We conducted a retrospective review of lymphoma cases in children aged 0–17 years diagnosed at the Children’s Hospital of Mexico between 2004 and 2014. Cases were classified according to the ICCC-3 system and further described by histopathological subtype, age group, sex, and clinical outcomes. **Results**: Hodgkin lymphoma (HL) was the most frequent diagnosis, followed by non-Hodgkin lymphoma (NHL). Among HL cases, nodular sclerosis and mixed cellularity predominated, particularly in school-age children and adolescents. Within NHL, precursor T-cell lymphoma represented the largest subgroup, whereas mature B-cell lymphomas, such as diffuse large B-cell lymphoma, were less common than typically reported in high-income settings. Burkitt lymphoma occurred mainly among younger children. HL showed high survival, while some NHL subtypes exhibited poorer outcomes. **Conclusions**: This large hospital-based case series provides characterization of pediatric lymphomas in a major Mexican referral center. While HL subtype patterns resembled global trends, the predominance of precursor T-cell lymphomas within NHL contrasts with observations from high-income regions. These findings highlight the value of institutional case registries and the need for more comprehensive outcome reporting in future studies.

## 1. Introduction

Lymphomas, including both Hodgkin (HL) and non-Hodgkin (NHL) subtypes, represent the third most prevalent childhood malignancies [1]. Their prevalence, however, varies considerably by region. In the Mediterranean region, lymphomas represent the most common cancer in individuals aged 15–19 years [2]. In the United States, data from the Surveillance, Epidemiology, and End Results Program indicate that these malignancies account for approximately 15% of all childhood cancers [3]. In Mexico, childhood lymphomas rank as the third most common pediatric malignancy, comprising approximately 11.1% of all cancers diagnosed in children younger than 15 years [4].

Overall, childhood lymphomas show a male predominance, with varying male-to-female ratios across subtypes [3,5,6]. The etiology of pediatric lymphomas is multifactorial and incompletely understood. Infectious, immunological, and environmental factors have been implicated in lymphomagenesis [7,8,9]. Among these, Epstein–Barr virus (EBV) infection has a well-established association with several lymphoma subtypes, including HL—particularly mixed cellularity and lymphocyte-rich variants—as well as Burkitt lymphoma (BL) and certain T/NK-cell lymphomas [9,10]. The prevalence and timing of primary EBV infection vary across geographic regions and socioeconomic contexts, which may contribute to observed differences in lymphoma subtype distribution worldwide [11].

These neoplasms are characterized by the abnormal proliferation of lymphoid cells and typically present with lymphadenopathy, most commonly involving the cervical, mediastinal, supraclavicular, or axillary regions. Other frequent clinical manifestations include persistent fever, night sweats, unexplained weight loss, and pruritus [12,13]. Diagnosis is primarily established through histopathological evaluation of lymphoid tissue, usually obtained via excisional biopsy.

The International Classification of Childhood Cancer, Third Edition (ICCC-3), is a pivotal framework for the diagnosis, treatment, and research of lymphomas in pediatric populations [14]. This system categorizes lymphomas into five primary groups based on histological type and clinical behavior: HL, NHL, BL, miscellaneous lymphoreticular neoplasms, and Unspecified lymphomas [14].

In this study, we aim to describe a case series of pediatric lymphoma treated at the Children’s Hospital of Mexico Federico Gómez (CHMFG) during 2004–2014.

## 2. Materials and Methods

### Study Population

We included all pediatric lymphoma cases diagnosed at the CHMFG between 2004 and 2014. Only cases confirmed by a certified pathologist through histological and immunohistochemical evaluation of biopsy specimens were eligible. Cases of precursor B-cell and T-cell lymphomas were included only when the percentage of bone marrow blasts was less than 20%, ensuring exclusion of leukemia-equivalent cases according to standard criteria. Cases were classified according to the ICCC-3 system [14] and were further stratified by histopathological subtypes for descriptive analysis.

Although diagnostic criteria and referral patterns have evolved over the past decade, the histopathological classifications used in this study align with internationally accepted standards at the time and remain comparable to the current ICCC-3 categories. Despite being historical, these data remain clinically relevant, as they constitute one of the most comprehensive pediatric lymphoma cohorts from a national referral center in Mexico.

Information on key demographic and clinical characteristics—including year of diagnosis, age at diagnosis, and sex—was available for all included cases. In contrast, outcome data (vital status) were available only for a subset of patients due to incomplete follow-up. The CHMFG is a national tertiary-level referral center located in Mexico City that provides care to pediatric patients without social security coverage from across the country. This population faces substantial socioeconomic barriers, and in several instances, families discontinue follow-up because they cannot afford repeated travel or temporary relocation to complete treatment until discharge. These circumstances explain the limited completeness of outcome data for some lymphoma subtypes. The 2004–2014 period represents the most internally consistent and fully validated segment of the institutional pathology registry. Post-2014 transitions in electronic medical record systems and reporting workflows generated partially incomplete and non-harmonized datasets, precluding their integration into a retrospective analytic framework.

Because CHMFG is a national referral center without a defined catchment population and receives patients from multiple states, an accurate population denominator is unavailable. Therefore, incidence and age-standardized rates were not calculated.

This study was approved by the Ethical and Scientific Review Boards of the CHMFG (Registry HIM-2016-089).

## 3. Results

### 3.1. Classification of Pediatric Lymphoma Cases

We analyzed 196 pediatric lymphoma cases diagnosed at the CHMFG over the 11-year period from 2004 to 2014. Cases were classified according to the ICCC-3 system [14] and were further stratified by histopathological subtypes for descriptive analysis. HL accounted for 43.4% of cases, followed by NHL at 40.8%. BL accounted for 11.2% of cases, while a small proportion (4.6%) corresponded to unspecified lymphomas. No miscellaneous lymphoreticular neoplasms were identified in this cohort (Table 1).

Among HL, the classical subtypes nodular sclerosis (42.7%) and mixed cellularity (41.5%) were the most frequent histological variants, whereas the lymphocyte-rich and lymphocyte-depleted subtypes were less common. Nodular lymphocyte-predominant accounted for 3.5% of all HL cases.

Within NHL, precursor T-cell lymphomas were the most frequent subtype (33.8%), followed by anaplastic large cell lymphoma (ALCL, 26.3%). Diffuse large B-cell lymphoma (DLBCL) accounted for a smaller but relevant proportion of cases (18.8%), while precursor B-cell lymphomas represented 11.3%. Cutaneous T-cell lymphomas and extranodal NK/T-cell lymphoma, nasal type, were uncommon (Table 1).

The overall annual number of pediatric lymphoma cases diagnosed over the 11-year study period ranged from 10 to 26 cases per year. Year-to-year variability was observed, with no consistent increasing or decreasing temporal trend. Given the small number of cases per year, no further subgroup-specific temporal analyses were performed.

### 3.2. Sex Distribution Among Pediatric Lymphoma Subtypes

**Figure 1:**
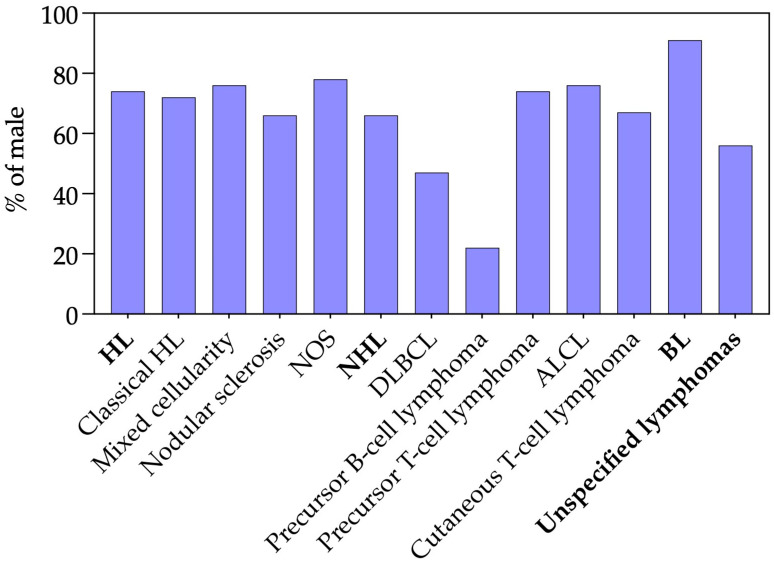
Male proportion across pediatric lymphoma subtypes. Bar plot showing the percentages of males per lymphoma subtype. Only subtypes with ≥6 cases are plotted. Overall, 71% of pediatric lymphoma cases involved males. HL: Hodgkin lymphoma, NOS: Not otherwise specified, NHL: non-Hodgkin lymphoma, DLBCL: Diffuse large B-cell lymphoma, ALCL: Anaplastic large cell lymphoma, BL: Burkitt lymphoma.

Among lymphoma subtypes with ≥6 cases, most exhibited a clear male predominance (Figure 1). HL were generally predominant in males, with classical, mixed cellularity, nodular sclerosis, and not otherwise specified (NOS) subtypes ranging from 66% to 78% male. NHL showed greater variability: DLBCL was roughly balanced between sexes, whereas precursor B-cell lymphoma was strikingly predominant in females. In contrast, T-cell subtypes—including precursor T-cell lymphoma, ALCL, and cutaneous T-cell lymphoma—were predominantly male, as were BL, which were overwhelmingly male (>90%). Overall, males accounted for 71% of pediatric lymphoma cases, highlighting a general male predominance across subtypes, with precursor B-cell lymphoma as a notable exception (Figure 1).

### 3.3. Age Distribution Across Pediatric Lymphoma Subtypes

**Figure 2:**
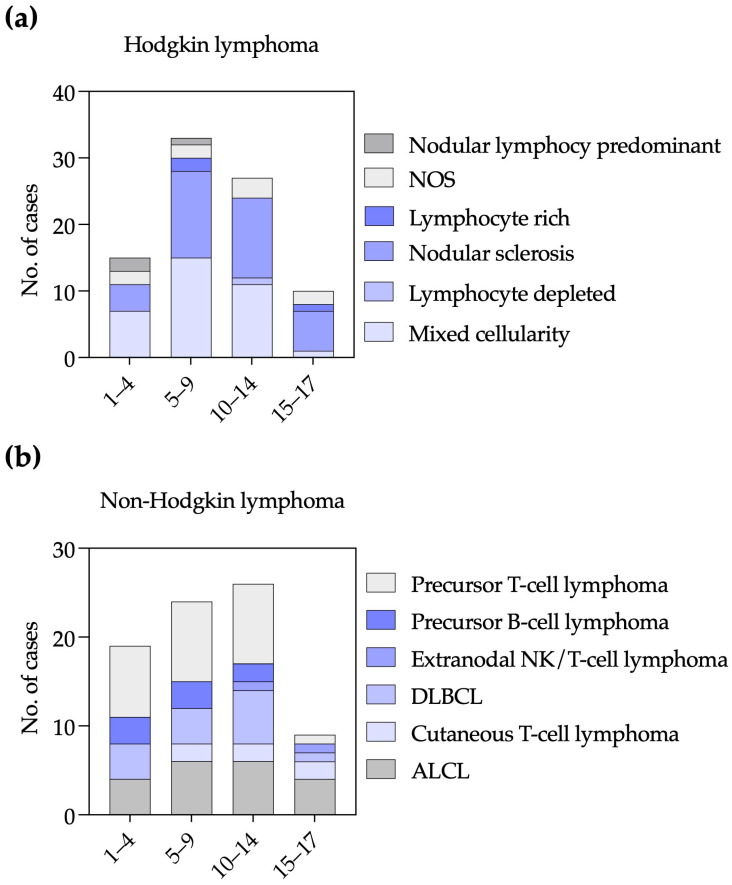
Age distribution of pediatric lymphoma subtypes. Bar plots showing the number of cases by age group for Hodgkin lymphoma (**a**), non-Hodgkin lymphoma (**b**). DLBCL: Diffuse large B cell lymphoma, ALCL: Anaplastic large cell lymphoma.

We analyzed the age distribution of lymphoma subtypes among children aged 1–17 years (Figure 2). For HL, nodular sclerosis and mixed cellularity were most frequent in the 5–9 and 10–14-year age groups, with markedly fewer cases in adolescents aged 15–17 years. Lymphocyte-rich HL was rare, with isolated cases in the 5–9- and 15–17-year groups. NOS cases were observed across all age ranges. Nodular lymphocyte-predominant HL was uncommon, occurring only in younger children (Figure 2a).

Among NHL cases, precursor T-cell lymphoma was the most common subtype in children aged 1–4, 5–9, and 10–14 years, with a sharp decline in adolescents aged 15–17 years. ALCL was more frequent in the 5–9 and 10–14-year age groups. DLBCL increased with age and peaked in the 10–14-year group. Cutaneous T-cell lymphoma was absent in children aged 1–4 years but present in all older groups. Precursor B-cell lymphoma occurred exclusively in children younger than 15 years. Extranodal NK/T-cell lymphoma, nasal type, was rare and restricted to older children and adolescents (Figure 2b). The small number of BL and unspecified lymphomas precluded meaningful analysis.

### 3.4. Mortality Across Lymphoma Subtypes

Outcome data were available for a substantial proportion of cases, varying by lymphoma subtype (see Methods). Mortality percentages were calculated only among patients with available outcome information (Table 2). Among patients with HL, overall mortality was low (4.8%). In contrast, mortality among NHL cases was higher (34.0%). BL showed intermediate mortality (26.7%), while mortality among unspecified lymphomas was 16.7%. It should be noted that outcome data were unavailable for a proportion of patients. Particularly among HL and NHL cases—the subtypes with the highest number of cases—mortality rates in patients lost to follow-up may be higher. For BL and unspecified lymphomas, the small number of cases precluded further interpretation.

## 4. Discussion

In this 11-year retrospective study, we describe the distribution of pediatric lymphoma cases diagnosed at the CHMFG, a major national referral center. Our analysis provides valuable insight into the patterns of lymphoma subtypes, demographic characteristics and clinical outcomes in a large pediatric cohort from Mexico.

To better situate our findings within the national landscape, we compared our results with the two largest sources of childhood lymphoma data available in Mexico. The population-based study by Rendón-Macías et al. [15], which analyzed children with social security coverage by the Mexican Social Security Institute (MSSI) in the Mexico City metropolitan area, reported a higher proportion of NHL (49.4%) than HL (37.2%). In contrast, our cohort—composed largely of uninsured patients treated at a national referral center—showed a predominance of HL (43%) over NHL (40.8%). These differences likely reflect the distinct populations served by each institution and the structure of the Mexican public health system, rather than true biological variation in lymphoma subtype distribution. Although both the MSSI and the CHMFG are national referral centers with access to specialized diagnostic services, they care for pediatric groups with different sociodemographic profiles, referral pathways, and patterns of clinical access, which may influence the case mix observed. In our cohort, delayed access to care among socioeconomically vulnerable patients may have contributed to selection bias, potentially leading to underdiagnosis of certain mature B-cell lymphomas. Advanced disease presentation in this setting could, in some cases, favor classification as leukemia rather than lymphoma, thereby influencing the relative frequencies observed in our hospital-based series.

In addition, differences in age ranges included in each dataset may also contribute: the IMSS study evaluated children younger than 15 years, whereas our series includes patients up to 17 years of age. Because NHL is relatively more common in younger children and HL increases in frequency during adolescence, this variation in age structure could partially influence the observed HL:NHL proportions. Taken together, these factors provide a plausible explanation for the contrasting subtype distributions across institutions.

National data provide an additional point of reference. The 2024 cross-sectional report from the Child and Adolescent Cancer Registry (CACR)—although limited to a single year—shows a distribution that closely mirrors our cohort: HL accounted for 45% of newly reported lymphomas, followed by NHL (43%) and a small fraction of BL (9%) [16]. While these registry data do not distinguish cases by type of health-care coverage, they nonetheless provide a nationwide snapshot of lymphoma patterns in Mexico and further highlight that HL can represent a substantial proportion of pediatric lymphomas at the national level. In Mexico, approximately 26.5 million children and adolescents (0–19 years)—nearly 60% of this age group—lack social security coverage [17]. By describing lymphoma patterns in an uninsured group, our study adds information that complements existing datasets focused largely on children with social security.

An additional strength of our study is the diagnostic granularity achieved. Neither the Rendón-Macías et al. report [15] nor the CACR data [16] include histologic subtyping of HL or NHL. By providing histological subtype-level classification, our analysis addresses this limitation and offers a more nuanced understanding of lymphoma patterns in this pediatric population. In particular, the distribution of NHL subtypes in our cohort differs from patterns typically observed in high-income countries: precursor T-cell lymphoma was the most common subtype, exceeding the frequency of DLBCL, which predominates in high-income settings [18]. Similar trends have been described in other middle-income regions and may reflect environmental, infectious, or genetic factors that shape regional lymphoma epidemiology [18,19]. BL accounted for 11% of all lymphomas, a frequency consistent with reports from non-endemic areas [20].

Taken together, our findings provide a complementary perspective on pediatric lymphoma in Mexico, particularly among uninsured children, a population underrepresented in national epidemiologic reports.

A male predominance was observed across most lymphoma subtypes, consistent with previous pediatric studies and likely reflecting sex-based differences in immune function or exposure-related factors [3,5,6,21]. An exception was precursor B-cell lymphoma, which showed a female predominance in our cohort; however, given the small number of cases, this finding should be interpreted with caution.

Mortality differed markedly between HL and NHL. HL showed low mortality, in line with its well-established favorable prognosis [22,23,24], whereas NHL displayed more heterogeneous outcomes across subtypes. These estimates require cautious interpretation due to incomplete outcome data but highlight the variability in clinical behavior among pediatric lymphomas.

A key strength of this study is the systematic classification of a relatively large single-institution pediatric lymphoma cohort using standardized ICCC-3 criteria [14], including detailed histological subtyping. This level of diagnostic resolution, together with the characterization of age and sex distributions, adds valuable information to the limited literature on pediatric lymphomas in Mexico.

## 5. Study Limitations

Several limitations of this study should be acknowledged. First, this is a single-institution case series with a limited sample size, which is insufficient to provide robust epidemiologic estimates of childhood lymphoma at the population level. As CHMFG is a national referral center without a defined catchment population, the data cannot be used to derive incidence rates or to draw firm conclusions about the epidemiology of pediatric lymphomas in Mexico. Second, outcome data were incomplete, with nearly one-third of patients lost to follow-up, which limits the strength of survival-related inferences and requires cautious interpretation of mortality findings. Third, some histological subtypes were represented by small numbers of cases, reducing the reliability of subtype-specific analyses.

## 6. Conclusions

Despite these limitations, our study provides an updated and detailed overview of pediatric lymphoma patterns at a major referral center in Mexico. The distribution of subtypes, and demographic characteristics closely aligns with global trends, while certain observations—such as the predominance of precursor T-cell lymphomas and the strong male bias—highlight the importance of conducting regional studies. Future work integrating multi-center data and improving outcome reporting will be essential for developing a more comprehensive picture of pediatric lymphoma epidemiology in Mexico.

## Figures and Tables

**Table 1:** Distribution of lymphomas in the Children’s Hospital of Mexico for the period 2004–2014.

ICCC-3 Group	Subtype	n	%
**IIa. Hodgkin lymphomas**		**85**	**43.4**
	Classical Hodgkin lymphoma	82	96.5
	Nodular sclerosis	35	42.7
	Mixed cellularity	34	41.5
	Lymphocyte-rich	3	3.7
	Lymphocyte-depleted	1	1.2
	Not otherwise specified	9	11.0
	Nodular lymphocyte-predominant HL	3	3.5
**IIb. Non-Hodgkin lymphomas**		**80**	**40.8**
IIb.1 Precursor cell lymphomas			
	Precursor B-cell lymphoma	9	11.3
	Precursor T-cell lymphoma	27	33.8
IIb.2 Mature B-cell lymphomas (except Burkitt)			
	Diffuse large B-cell lymphoma	15	18.8
IIb.3 Mature T/NK-cell lymphomas			
	Anaplastic large cell lymphoma	21	26.3
	Cutaneous T-cell lymphoma	6	7.5
	Extranodal NK/T-cell lymphoma, nasal type	2	2.5
**IIc. Burkitt lymphoma**		**22**	**11.2**
**IIe. Unspecified lymphomas**		**9**	**4.6**
Total		196	100

**Table 2:** Mortality across lymphoma subtypes. Mortality percentages were calculated only among patients with available outcome information.

Lymphoma Subtype	n	Alive (n)	Dead (n)	NI ^1^ (n)	Data Completeness (%)	Mortality (%)
Hodgkin lymphomas	85	59	3	23	72.9	4.8
Non-Hodgkin lymphomas	80	33	17	30	62.5	34.0
Burkitt lymphoma	22	11	4	7	68.2	26.7
Unspecified lymphomas	9	5	1	3	66.7	16.7

^1^ No information.

## Data Availability

The original contributions presented in this study are included in the article. Further inquiries can be directed to the corresponding author.

## Abbreviations

The following abbreviations are used in this manuscript:

HL	Hodgkin lymphoma
NHL	Non-Hodgkin lymphoma
BL	Burkitt lymphoma
CHMFG	Children’s Hospital of Mexico Federico Gómez
ALCL	Anaplastic large cell lymphoma
DLBCL	Diffuse large B-cell lymphoma
NOS	Not otherwise specified
